# Adaptive deep brain stimulation targeting the subthalamic nucleus in a patient with Parkinson’s disease: A CARE compliant case report

**DOI:** 10.1097/MD.0000000000044130

**Published:** 2025-08-22

**Authors:** Tirath Patel, Christopher Hanani, Anaya Noor, Shamikha Cheema, Muhammad Farhan, Rohab Sohail, Abhishek Goyal, Jabez David John, Adekunle E. Omole, Richard M. Millis

**Affiliations:** a Department of Neurology, Trinity Medical Sciences University School of Medicine, Saint Vincent and the Grenadines; b Department of Neurology, Henry Ford Health, Detroit, MI; c Department of Internal Medicine, Dow Medical College, Dow University of Health Sciences, Karachi, Pakistan; d Department of Internal Medicine, King Edward Medical University, Lahore, Pakistan; e Department of Internal Medicine, Ajman University College of Medicine, Ajman, United Arab Emirates; f Department of Internal Medicine, Bayhealth Medical Center, Dover, DE; g Department of Neurology, JFK University Medical Center, Edison, NJ; h Department of Internal Medicine, Malla Reddy Institute of Medical Sciences, Hyderabad, India; i Department of Anatomy, Louisiana State University Health Sciences Center, New Orleans, LA; j Department of Pathophysiology, American University of Antigua, Osbourn, Antigua and Barbuda.

**Keywords:** adaptive deep brain stimulation, dyskinesia, Parkinson’s disease, tremor

## Abstract

**Rationale:**

Adaptive deep brain stimulation (aDBS) represents a notable advancement in treating Parkinson’s disease (PD), as it offers enhanced therapeutic outcomes and personalized management by adjusting stimulation parameters in real-time according to individual neural signals. This approach minimizes adverse effects commonly associated with standard continuous deep brain stimulation (cDBS). This case report describes the progress of a 62-year-old man with severe PD who demonstrated notable enhancement in motor symptoms and quality of life throughout a 3-month trial period using aDBS.

**Patient concerns:**

A 62-year-old man who has been suffering from Parkinson’s disease.

**Diagnoses:**

The patient had been diagnosed with PD for 10 years. The patient’s motor symptoms, including dyskinesia during the on-state and akinesia during the off-state, progressively worsened over time.

**Interventions:**

The patient underwent bilateral subthalamic nuclei DBS surgery with cDBS. Following progressive worsening of motor symptoms, he was transitioned to aDBS.

**Outcomes:**

The aDBS system adaptively modified stimulation parameters by utilizing real-time neural feedback from beta band activity detected in the subthalamic nucleus, resulting in decreased dyskinesia and reduced reliance on medication. The customized strategy led to a significant improvement in motor symptoms, a reduction in dyskinesia, and an overall enhancement in quality of life during the 3-month trial period.

**Lessons:**

Existing evidence highlights the ability of aDBS to improve motor control and reduce problems associated with DBS, such as speech and gait abnormalities. Research findings have demonstrated significant improvements in motor scores and a reduction in stimulation time, highlighting the effectiveness of aDBS and its ability to prolong the lifespan of devices.

## 1. Introduction

Parkinson’s disease (PD) is a degenerative neurological condition that worsens over time. It is characterized by motor symptoms, including tremors, stiffness, bradykinesia, and postural instability.^[1]^ While levodopa is often considered the most effective treatment for PD, its long-term effectiveness is hindered by the emergence of motor problems such as dyskinesia and motor fluctuations. Deep brain stimulation (DBS) is considered a practical option for treating individuals with PD who experience movement problems, as suggested by Tinkhauser et al.^[2]^ Adaptive deep brain stimulation (aDBS) is an innovative method that dynamically utilizes brain signal feedback to adjust real-time stimulation parameters. This is in contrast with continuous Deep Brain Stimulation (cDBS), which delivers continuous fixed stimulation without any adjustments. According to Nakajima et al,^[3]^ aDBS has the potential to be linked to a reduced occurrence of long-term adverse effects caused by stimulation, such as dyskinesia or speech impairment. Multiple studies have demonstrated the feasibility and efficacy of aDBS in individuals with Parkinson’s disease, resulting in improvements in motor symptoms and quality of life and a decrease in side effects and medication use.^[1,4–6]^ Although aDBS shows promising results and has the potential to transform DBS therapy for PD, this technology is still in its early stages and requires additional research, as described in the Section 3.

## 2. Case report

**Figure 1.**
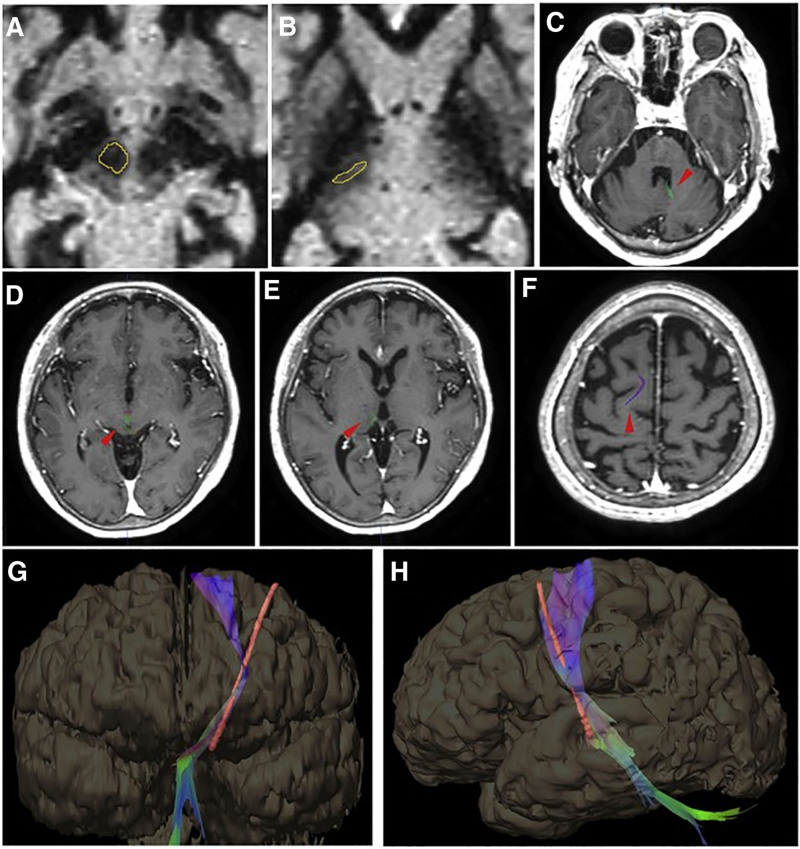
Two regions of interest (A) red nucleus and (B) ventral intermediate nucleus are illustrated using the software’s brush application. The dentate-rubro-thalamic tract on axial T1-weighted images at the level of the dentate nucleus (C), the red nucleus (D), the thalamus (E), and the precentral gyrus (F). Images of the fiber tract and the DBS (deep brain stimulation) electrode reconstructed in 3D (G and H). The photos are collected from the first case. DBS = deep brain stimulation.

A 62-year-old male patient who has been suffering from Parkinson’s disease for 10 years was sent to the hospital for a deep brain stimulation (DBS) examination. This was due to inadequate management of motor symptoms and side effects caused by medications. The patient had been undergoing levodopa therapy for an extended period and was currently encountering the phenomenon of “wearing off” and dyskinesia. The individual exhibited motor symptoms, including bradykinesia, stiffness, and tremors on both sides, with more severe symptoms on the right side. After a comprehensive assessment, the patient was determined to be ideal for DBS surgery. The target for stimulation was the bilateral subthalamic nucleus (STN; Fig. 1). The patient underwent a bilateral STN-DBS implantation, which was performed successfully under general anesthesia. The stimulation settings were set as follows: frequency, 130 Hz; pulse width, 60 µs; and amplitude, 2.5 V. During the initial months after surgery, the patient exhibited a substantial improvement in motor symptoms and overall quality of life. Over a period of 9 months, the patient started to experience variations in their reaction to stimulation, and their dyskinesia worsened. The patient stated that his symptoms had exacerbated during the afternoon and night.

**Figure 2.**
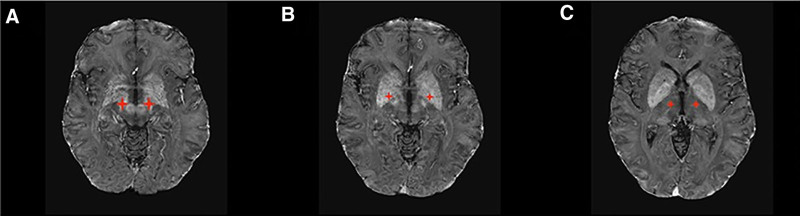
DBS (deep brain stimulation) lesions.

To resolve these concerns, it was determined that the patient should be switched back to aDBS. The patient underwent further imaging to verify the placement of DBS leads, and an implantable neurostimulator capable of aDBS was implanted. The aDBS device employs a feedback algorithm that relies on beta band activity captured from the STN electrodes to modify the stimulation parameters dynamically. Subsequently, the patient underwent a 3-month trial of aDBS, as shown in Figure 2. The aDBS system modified the stimulation parameters throughout this period by considering beta-band activity. As a result, there was a decrease in the overall duration of stimulation and an enhancement in motor performance. The patient showed decreased dyskinesias and variations in response to stimulation. Following the 3-month trial period, the patient underwent a comprehensive evaluation of their motor function using the Movement Disorder Society-Unified Parkinson’s Disease Rating Scale, a standardized tool for assessing the severity and progression of Parkinson’s disease symptoms. The patient’s overall MDS-UPDRS score significantly improved from 70 to 50, alongside a decrease in the motor subscore from 42 to 28. Additionally, the patient completed the Parkinson’s Disease Questionnaire-39, which revealed elevated scores. The patient thereby noted an enhancement in their quality of life, accompanied by a decrease in the duration of motor activities required for everyday functioning.

## 3. Discussion

cDBS involves continuous stimulation delivery, which can result in a range of adverse effects and suboptimal therapeutic outcomes; however, aDBS is a novel method that adjusts stimulation parameters based on current neural activity. This personalized approach offers a more effective treatment option.^[5,7]^ This report focuses on a 62-year-old male with advanced Parkinson’s disease for whom a transition was made to aDBS following an unsatisfactory response and notable adverse effects of the initial cDBS treatment. The customized strategy resulted in significant improvements in motor symptoms and overall quality of life, as indicated by elevated scores on the Parkinson’s Disease Questionnaire-39 for this patient and in various other cases.^[8,9]^

Recent research has emphasized the possibility that aDBS dramatically improves motor function in patients with Parkinson’s. A pivotal investigation demonstrated a remarkable 66% enhancement in motor scores during unblinded evaluations and a 50% improvement during blinded evaluations, which is notably superior to the outcomes found with cDBS. These improvements were achieved by reducing the stimulation time by 56%, indicating more effective power utilization and potentially extending the device’s lifespan.^[1]^ Studies on bilateral aDBS have shown significant enhancements in axial and limb symptoms, highlighting the system’s ability to accurately adjust stimulation parameters throughout the day and under different drug conditions. The ability to adapt therapy according to changing conditions commonly observed in Parkinson’s disease care is essential. Moreover, research has demonstrated that employing a dual-target stimulation approach, which targets both the STN and the globus pallidus, is more effective in alleviating motor symptoms than stimulating each region separately. This highlights the potential of aDBS to provide more comprehensive symptom management.^[9,10]^

Regarding the safety of aDBS, numerous studies have consistently demonstrated the absence of substantial adverse effects, thereby establishing it as a safer option than cDBS.^[1,9]^ Regarding optimization, identifying and utilizing biomarkers, such as Beta Band Power from the STN, is effective in guiding adjustments to stimulation. This helps ensure that therapy is closely linked to a patient’s clinical condition.^[5]^ Longitudinal studies are crucial for evaluating the long-term effects of aDBS. A significant 5-year longitudinal trial demonstrated enduring enhancements in motor symptoms. It decreases reliance on medication, highlighting the long-lasting advantages of STN DBS in treating Parkinson’s disease.^[11]^ Subsequent studies aimed to confirm these promising results in larger patient cohorts through randomized controlled trials to establish the superiority of aDBS over conventional therapies.^[11–16]^ In this case, the patient was successfully treated with aDBS, and significant improvements in motor function and quality of life were observed. No adverse effects were attributed to the overall treatment.

Despite these promising results, aDBS is still in the developmental phase and faces challenges in biomarker identification, designing systems that respond to real-time feedback, and conducting long-term safety evaluations. Future research should focus on large-scale studies and extended follow-up to validate and refine aDBS, enhancing its effectiveness and long-term viability as a treatment option for PD. By advancing our understanding and technology in aDBS, we can move closer to personalized brain stimulation treatments that significantly improve patient outcomes in Parkinson’s disease.

## 4. Conclusion

This case report highlights the potential of a specialized type of brain stimulation, known as aDBS, for managing advanced PD. It demonstrated significant improvements in motor function and quality of life in a 62-year-old male patient. By dynamically adjusting stimulation settings based on immediate feedback from brain activity, aDBS effectively reduces symptoms while minimizing the side effects typically associated with traditional cDBS, such as dyskinesia and reliance on medication.

## Acknowledgments

Additionally, the abstract of the case report was accepted for e-poster presentation at the North American Neuromodulation Society Annual Meeting in Orlando, Florida, 2025. This work was reported in line with the CARE criteria.^[17]^ The authors would also like to acknowledge the use of Grammarly and PaperPal to improve the grammatical accuracy and clarity of this manuscript, and no other external support was received for this study.

## Author contributions

**Conceptualization:** Tirath Patel.

**Data curation:** Rohab Sohail.

**Writing – original draft:** Tirath Patel, Christopher Hanani, Anaya Noor, Shamikha Cheema, Muhammad Farhan, Abhishek Goyal, Jabez David John, Adekunle E. Omole.

**Writing – review & editing:** Tirath Patel, Anaya Noor, Adekunle E. Omole, Richard M. Millis.
